# Comfort and utility of school-based weight screening: the student perspective

**DOI:** 10.1186/1471-2431-8-9

**Published:** 2008-03-03

**Authors:** Karrie A Kalich, Virginia Chomitz, Karen E Peterson, Robert McGowan, Robert F Houser, Aviva Must

**Affiliations:** 1 Health Science Department, Keene State College,229 Main Street, Keene, NH 03435-2903, USA; 2 Institute for Community Health, 119 Windsor St, Cambridge, MA 02139, USA; 3 Nutrition and Society, Human Development, and Health, Harvard School of Public Health, 677 Huntington Ave, Kresege 617, Boston, MA 02115, USA; 4 Health, Physical Education & Athletics, Cambridge Public Schools, 159 Thorndike Street, Cambridge, MA 02141, USA; 5 Friedman School of Nutrition Science and Policy, Tufts University, 150 Harrison Ave. Boston, MA 02111, USA; 6 Dept of Public Health and Family Medicine, Tufts University School of Medicine, 136 Harrison Ave., Boston, MA 02111, USA

## Abstract

**Background:**

Weight screening in schools has been proposed as one strategy to address childhood obesity. Students' response to such screening is unexplored, however. In this study we evaluated the perceived comfort, utility and impact of school-based weight screening from the perspective of middle school-aged students.

**Methods:**

A cross-sectional study of 852 ethnically diverse 5^th^–8^th ^grade students. Associations were investigated between measured height and weight screening data and responses to a self-administered questionnaire completed immediately following weight screening in physical education class. BMI categories were based on the revised 2000 CDC growth chart and definitions: 5^th^–85^th ^BMI percentile = healthy weight, 85^th^–95^th ^BMI percentile = at risk for overweight, and >95^th ^percentile BMI = overweight.

**Results:**

Overall, students' comfort level with weight screening varied depending on the student's own weight status. More overweight students (38.1%) reported being uncomfortable than healthy weight students (8.1%) (p < 0.001). In particular, overweight female students (54.8%) compared to healthy weight female students (21.6%) reported being uncomfortable (p < 0.01). About half (54.9%) of all students reported knowing their weight prior to screening, and 58.9% reported that it was useful to learn their height and weight. Compared to healthy weight students, overweight students were significantly more likely to report the intention to perform weight modification related activities such as visiting a doctor (Odds ratio (OR) = 2.0, 95% CI = 1.3, 3.1), eating more fruits and vegetables (OR = 2.7, 95% CI = 1.7, 4.1), and increasing physical activity (OR = 4.3, 95% CI = 2.7, 7.0).

**Conclusion:**

Overall, the majority of the middle school students did not report discomfort with school-based weight screening, did report that receiving height and weight information was useful, and generally report appropriate weight control intentions. These proportions varied across weight status categories, however, with students who were at risk for overweight or overweight reporting higher levels of discomfort. For schools that conduct weight screening, it is essential that they also provide comfortable and private settings as well as education or counseling regarding healthy weight control practices.

## Background

Epidemic levels of overweight children in the United States [1] have led school administrators to seek effective and appropriate ways to promote healthy weights in their students. The 2004 Institute of Medicine (IOM) report suggests that schools gather weight, height, and body mass index (BMI) measurements on students annually and that schools communicate the results of screening to parents [2]. This relatively new role for schools reflects concern over the substantial physical and psychosocial health risks associated with overweight in childhood and the likelihood that childhood obesity will persist into adulthood [1,3-5].

Even before the IOM report, several school systems in the U.S. had implemented school-based weight screening. Many school systems had been collecting student height and weight information or contemplating its collection [6-10] with the belief that sharing weight status information could increase family awareness of the child's weight status and would motivate the family to take appropriate actions to address the child's potential weight problem. Arkansas, an early adopter of this initiative, passed a law that required schools to weigh and measure students and to send a 'BMI report card' home to parents [6]. Several states such as Florida, Pennsylvania and Tennessee ([6,8] and [9]) also have legislation in place to support schools in conducting BMI screening. The early enthusiasm for school-based weight screening as a tool to help combat the obesity epidemic was tempered by concern about the potential for unintended negative consequences.

The debate over the appropriateness of student BMI screening and reporting to parents continues [11-15]. In early 2006, Arkansas reconsidered the appropriateness of their BMI screening mandate [16], and opted to continue state-wide BMI screening, but modified their approach. Currently Arkansas conducts weight screening in the even years from kindergarten through 10^th ^grade and parents are able to opt out of the screening protocol each year on behalf of their child (Personal Communication June 19, 2007-**Dr. Joseph Thompson**, Associate Professor, University of Arkansas for Medical Sciences and the Surgeon General for the State of Arkansas.) Also critical, but yet unexplored, is the students' assessment of the comfort and utility of school-based weight screening. Provision of a "comfortable" setting for weight screening could mitigate the potentially harmful effects of weight screening. A private and respectful environment may lessen peer "teasing" and "labeling" of overweight children and thus reduce negative behavioral outcomes, such as unhealthful weight management practices.

In 2005, the expert panel convened by the Centers for Disease Control to discuss the role of schools in addressing child overweight included BMI weight screening among the topics discussed. The committee concluded that evidence was insufficient to recommend that schools conduct BMI screening of students and report results to parents [15]. Some of their concerns related to the ability of schools to recommend effective, accessible and affordable therapies to families once a child was identified as overweight [15]. A comprehensive review of policies and research related to BMI screening [15] concluded that BMI screening is not necessary for obesity monitoring at the national level, although the practice could provide useful information for monitoring trends at the state and local level.

To date, two studies have investigated the acceptance of BMI screening from the parents' perspective [17-19]. Both studies found that, in general, parents were supportive of the practice and desired to receive the BMI screening information annually. Parents felt it was important to ensure that screening was private and respectful, and that results be provided to parents in a neutral, non-judgmental manner that avoided labeling [19].

In Cambridge, Massachusetts, a surveillance system based in the public schools' physical education (PE) department has been in place since 1999. PE teachers annually measure weight, height, and fitness levels on all elementary school students. We previously reported parental awareness and concern in response to our K-8 public school's health and fitness report card [17]. Parents who received personalized BMI report cards were more likely to be aware of their child's weight status compared to parents not receiving personalized information. Among the parents receiving their child's personalized BMI information, parents of overweight children were more likely to report the intention to seek medical support, as well to implement a diet [17]. Despite the theoretical and practical advantages of school-based weight screening, concerns that weight status information may encourage unsupervised restrictive dieting, disordered eating, or have negative psychological impact have surfaced [11-15].

Behavior change theory posits that creating awareness of the need to make lifestyle changes increases behavioral intention and gradually results in behavior change [20]. The underlying, but as yet untested, assumption is that by informing a child and/or parent of the child's at-risk for overweight or overweight status, they will seek confirmation by a health care professional and/or may initiate positive lifestyle changes to assist in the achievement of a healthier body weight. As more policy-making institutions recommend that school-aged children be screened for overweight [2,21], school administrators and policy makers need information on its impact.

The current study was designed to evaluate how 5th–8th grade students perceive the comfort and utility of school-based height/weight screening and to assess the impact of screening on their reported behavioral intention to initiate weight management-related activities.

## Methods

### Study Context

The study was designed to evaluate an existing weight and height screening protocol that was initiated in 1999 by the Physical Education (PE) department in Cambridge Massachusetts elementary public schools, which include grades Kindergarten-8^th ^grade (K-8). Every April, in all schools kindergarten through 8^th ^grade, PE teachers measure the height and weight of students during PE class; since 2001 weight screening is part of comprehensive weight and fitness report card program undertaken annually.

### Study Population and Protocol

In 2003, a convenience sample of seven ethnically diverse public schools in Cambridge, Massachusetts was selected; individual schools were invited to participate in the study based on their prior successful collaboration with the investigators. In total, principals from seven of the 14 public elementary (K-8) schools in Cambridge were invited and all agreed to participate. The study population included 986 5^th^–8^th ^grade students.

In April 2003, as in previous years, prior to annual screening, PE teachers attend a one-hour training session to review standard weight, measuring and recording techniques [22], and to receive guidance with regard to recognition of warning signs of eating disorders and approaches and/or resources to address them. To ensure privacy, each student was individually invited to a screening area where the PE teacher measured and weighed them. The PE teacher recorded the measured height and weight. All other students were engaged in activities on the opposite side of the gymnasium. For added privacy, floor scales were used so that only the PE teacher and the individual student could view the weight result. One PE teacher per school conducted the weight and height screening.

Height was measured with a stadiometer (Seca 216 Accu-Hite, Snoqualmie, WA) to the nearest quarter inch. Weight was measured to the nearest two tenths pound using calibrated, digital scales (Seca Corporation, Hamburg, Germany). Data are recorded on paper forms.

Students were informed of their height and weight, but not their BMI or weight status classification. The Cambridge Public School District shares weight screening classifications with the students' caregivers by mail, together with locally available resources. This approach was adopted by school district administration to give the caregivers' the opportunity to share the weight status results (or not) with their child in the manner they deem appropriate.

The 2003 Student Survey was administered immediately following the measurement of height and weight. Members of the research team invited students to complete the survey and directed them to a location on the gym floor that was a minimum of five feet apart in all directions from other students also completing the survey. The students were provided with the survey, a clipboard and pencil. A second research team member monitored the students to ensure each student completed the survey without interference from their classmates. The completed surveys were given directly to a research team member upon completion. The self-administered survey was completed individually by all students, except for the 23(2.3%) students whose parents had opted out on their behalf and those who were absent (n = 111) from school on the day their class was scheduled for weight screening; no students refused to complete the survey. In all, 852 were surveys were completed.

To provide adequate human subjects protection, the study protocol included: 1) a notification letter (in English, Spanish and Haitian Creole) sent to the child's home address that described the survey research and provided a mechanism for caregivers to indicate in writing if they did not wish their child to participate in the survey, and 2) at the time of administration, notifying students their participation in the survey was voluntary. The study protocol for the evaluation of school-based weight screening was approved by the Institutional Review Board of the Cambridge Health Alliance.

### Measures and Variable Definition

A 27-question, self-administered survey to assess students' perceived comfort, utility, and behavioral intention as a result of screening was developed for the study. The survey instrument was pre-tested with seven 10-year olds who were prompted to provide feedback on confusing questions. The final survey instrument is available upon request. The original survey in-part utilized validated questions from the Harvard School of Public Health Food and Activity Survey and the Centers for Disease Control Youth Risk Behavioral Survey. The items were reviewed for content and face validity by researchers with expertise in child weight issues and school health. Comfort was measured by items that assessed: adequate privacy, comfort level with weighing in general and in PE class, and indicated the locations at which students felt height and weight should be measured. The five-point Likert-type responses were dichotomized into uncomfortable ("slightly uncomfortable" or "very uncomfortable") and not uncomfortable ("very comfortable," "slightly comfortable," or "neither comfortable, nor uncomfortable"). Utility was measured by: prior knowledge of height and weight, reported regular tracking of height and weight, and reported usefulness of learning height and weight. Behavioral intention was assessed based on the reported likelihood (on a 5-point Likert-type scale) to perform 11 different weight-management related activities. Students were asked "Based on receiving your height and weight information, how likely or not are you to do any of the following?" The five-tiered responses were dichotomized into likely ("very likely" or "slightly likely") and not likely ("slightly unlikely," "very unlikely," or "neither likely, nor unlikely").

Age was calculated based on the student's reported date of birth. BMI (kg/m^2^) was calculated from measured values. Race/ethnicity categories were self-identified as Non-Hispanic white, Non-Hispanic black, Hispanic, Asian, or other. Of the 852 students surveyed, 54 (6.3%) were missing one or more variables required to determine their BMI classification which reduced the sample size to 798.

### Data Analyses

Using SAS code developed by the CDC, weight, height, gender, and age measured in months was converted to BMI-for-age z scores [23]. No BMIz scores fell outside of the CDC's criteria for excluding biologically implausible BMIz scores [24]. As recommended by Barlow and Dietz [25], weight status categories were defined as: underweight (BMIz scores ≤ -1.645 (5^th ^percentile), healthy weight (-1.645 < BMIz score ≤ 1.036 (85^th ^percentile), at-risk for overweight (1.036 < BMIz score < 1.645 (95^th ^percentile), and overweight (BMIz scores ≥ 1.645).

Descriptive statistics for comfort, utility and behavioral intention measures were generated for the overall sample and by gender and weight status. Differences by age, gender, and weight status were evaluated with chi-square tests. Multivariate logistic regression analysis was performed to assess the relation between comfort with weight screening and weight status while controlling for gender, age, and race/ethnicity. We also used logistic regressions to assess the relation between the intention to perform a specific weight-related activity as a result of learning height and weight while controlling for gender, age, race/ethnicity. The statistical significance of the linear trend across weight status categories was assessed by a Chi-square test for linear trend, and implemented using StatCalc (a module of EpiInfo™ software, CDC, Atlanta, GA). Second order interactions (gender by weight status, gender by ethnicity, and weight status by ethnicity) were evaluated. All analyses (except for linear trend analyses) were accomplished using SPSS Version 12.0 for Windows (Chicago, Illinois).

## Results

Of the 986 students who were eligible for the study, 23 opted out of the study, 111 were absent on the day of their class was scheduled for weight screening, 54 were missing one or more variables required to determine their BMI classification and 12 underweight subjects were excluded from the study analyses because our interest was in the comparison between healthy weight and overweight and there were too few students in this category to consider it separately. Thus, our analytic sample comprises 786 students.

### Description of Study Population

The mean (SD) age of the 786 students with measured height and weight, and survey data was 12.0 (+ 1.2) years. Overall, 15.6% (95% CI 11.2, 17.8) of males and 18.3% (95% CI 14.4, 22.2) of females were at-risk for overweight, and 20.8% (95% CI 15.5, 22.9) of males and 16.4% (95% CI 12.0, 19.0) of females were overweight. The highest prevalence of overweight was observed among Hispanic males (35.0%) and non-Hispanic black females (24.6%) (Table 1).

**Table 1:** Demographic characteristics of students by Weight Status Category^1^

	**Overall^2^** **n = 786** **n**	**Healthy Weight** **n = 506** **%(n)**	**At-risk for Overweight** **n = 133** **%(n)**	**Overweight** **n = 147** **%(n)**
**Gender^3^**				
Overall	786	64.4(506)	16.9(133)	18.7(147)
Male	409	63.6(260)	15.6(64)	20.8(85)
Female	377	65.3(246)	18.3(69)	16.4(62)

**Age (years)**				
Males				
10 and under	50	11.9(31)	12.5(8)	12.9(11)
11	94	23.5(61)	23.4.(15)	21.2(18)
12	108	26.2(68)	28.1(18)	25.9(22)
13	114	26.9(70)	26.6(17)	31.8(27)
14 and older	43	11.6(30)	9.4(6)	8.2(7)
				
Females				
10 and under	55	15.8 (39)	8.7(6)	16.1(10)
11	89	23.6 (58)	20.3(14)	27.4(17)
12	102	24.8 (61)	31.9(22)	30.6(19)
13	97	26.0 (64)	30.4(21)	19.4(12)
14 and older	34	9.7 (24)	8.7(6)	6.5(4)

**Race ethnicity**				
**Males**				
Non-Hispanic white	162	67.9(110)	13.0(21)	19.1(31)
Non-Hispanic black	122	61.5(75)	17.2(21)	21.3(26)
Hispanic	40	42.5(17)	22.5(9)	35.0(14)
Asian	33	72.7(24)	18.2(6)	9.1(3)
Other	50	68.0(34)	12.0(6)	20.0(10)
				
**Females**				
Non-Hispanic white	134	71.6(96)	16.4(22)	11.9(16)
Non-Hispanic black	114	53.5(61)	21.9(25)	24.6(28)
Hispanic	43	67.4(29)	14.0(6)	18.6(8)
Asian	31	87.1(27)	6.5(2)	6.5(2)
Other	52	57.7(30)	26.9(14)	15.4(8)

^1^Weight status definitions based on CDC 2000 Growth Reference (16): Healthy weight = 5^th ^to <85^th ^percentile BMI; at-risk for overweight = 85^th ^to <95^th ^percentile BMI; Overweight = ≥95^th ^percentile BMI.

^2 ^Sample sizes vary slightly due to missing data.

^3 ^Percentages sum to 100% row-wise.

#### Comfort level and Adequacy of Privacy

Approximately 15% of students reported being slightly or very uncomfortable with having their weight measured at school (Table 2). All weight status groups felt more comfortable having their height measured than their weight. For each category of weight status, females were more likely than males to report feeling slightly uncomfortable or very uncomfortable (21.6% and 8.9% respectively, Χ^2 ^= 31.8, p =< 0.001). Few of the healthy weights students (8.1%) were uncomfortable with weight screening compared with those who were at risk for overweight (15.9%) or overweight (38.1%), Χ^2 ^= 75.7, p < 0.01).

**Table 2:** Student report of lack of privacy and discomfort^1 ^with weight screening by weight status^2 ^and gender'

		**Males**					**Females**				
	
	**Overall^3 ^Both genders** **N = 784**	**Overall** **n = 406**	**Healthy Weight** **n = 258**	**At-risk for overweight** **n = 63**	**Overweight** **n = 85**	**P for trend^4^**	**Overall** **n = 377**	**Healthy Weight n = 246**	**At-risk for overweight N = 69**	**Overweight n = 62**	**P for trend**
**% slightly or very uncomfortable with school-based weight screening**	14.9	8.9	3.9	6.3	25.8	<0.01	21.6	12.4	24.6	54.8	<0.01
**% slightly or very uncomfortable with height screening**	6.8	5.4	3.9	1.6	12.9	<0.01	8.2	8.9	4.4	9.7	0.830
**% slightly or very uncomfortable with weight screening in general**	19.4	11.3	5.4	6.4	33.3	<0.01	28.1	17.5	36.2	61.3	<0.01
**% reported enough privacy with screening (%yes)**	80.1	80.3	82.6	80.6	72.9	0.064	80.1	84.4	80.9	61.3	<0.01

^1 ^The percentages reflect the percentage of respondents who reported being "very uncomfortable" or "slightly uncomfortable" on a 5-point Likert scale.

^2 ^Weight status definitions based on CDC 2000 Growth Reference (16): Healthy weight = 5^th ^to <85^th ^percentile BMI; At-risk for overweight = 85^th+ ^to <95^th ^percentile BMI; Overweight = ≥95^th ^percentile BMI.

^3^Sample sizes vary slightly due to missing data.

^4^Significance testing based on chi-square test for linear trend.

More than half of overweight females (54.8%) reported feeling slightly uncomfortable or very uncomfortable. No differences in comfort with screening were observed across age groups.

Most students (80.1% overall) felt privacy was adequate (Table 2), with little difference by gender. Fewer overweight students (72.9% of males and 61.3% of females) reported adequate privacy than students of other weight status categories. Female students of healthy weight (84.4%), were more likely to report adequate privacy compared to at-risk for overweight (80.9%) and overweight (61.3%), Χ^2 ^= 14.7, p < 0.01).

With multivariate adjustment for age, gender, and race/ethnicity, students classified as at-risk for overweight were 2.6 times (95% CI 1.4, 4.8) as likely and overweight students were 8.3 (95% CI 4.8, 14.2) times as likely to report being uncomfortable with weight screening as students classified as healthy weight. Female students were 3.7 (95% CI = 2.3, 6.1) times as likely to report being uncomfortable as male students. Racial/ethnic category was not related to reported comfort. Gender by weight status interactions were not statistically significant.

Students could select more than one place where they thought that weight screening should be performed (Table 3). Overall, 30.6% of students identified a school setting (physical education class 21.8% and/or school nurse's office 14.7%) as suitable venues. The doctor's office was most frequently endorsed for weight screening, with two-thirds (66.2%) of students indicating it was suitable. Only 2.0% of students indicated that weight screening should occur "not at all."

**Table 3:** Percentage of students indicating weight screening location^1 ^is appropriate, by weight status category^2 ^(% yes (n)).

		**Males**					**Females**				
	**Overall^3 ^Both genders** **n = 784** **%(n)**	**Overall** **n = 409** **%(n)**	**Healthy Weight** **n = 260** **%(n)**	**At-risk for overweight** **n = 64** **%(n)**	**Overweight** **n = 85** **%(n)**	**P for trend^4^**	**Overall** **n = 375 %(n)**	**Healthy Weight** **n = 244** **%(n)**	**At-risk for overweight** **n = 69** **%(n)**	**Overweight** **n = 62** **%(n)**	**P for trend**

**Physical Education Class**	21.8(182)	27.4 (118)	30.8 (78)	24.2 (15)	19.0(16)	0.027	16.1 (64)	17.2 (42)	20.3 (14)	9.7(6)	0.282
**School Nurse's Office**	14.7(120)	14.1 (61)	16.6(42)	9.7(6)	11.8(10)	0.211	14.9 (59)	17.6(43)	10.1(7)	11.3(7)	0.116
**Doctor's Office**	66.2(553)	62.0 (268)	64.4(163)	54.8(34)	63.5(54)	0.841	71.6 (285)	72.7(178)	65.2(45)	72.6(45)	0.660
**At Home**	23.1(191)	23.6 (102)	26.9(68)	19.4(12)	20.0(17)	0.175	22.4 (89)	21.6 (53)	30.4(21)	16.1(10)	0.717
**Not at all**	2.0(19)	3.0 (13)	2.8(7)	-^5^	-	-	1.5 (6)	-	-	0.0(0)	-

^1 ^Respondents could select more than one location.

^2 ^Weight status definitions based on CDC 2000 Growth Reference (16): Healthy weight = 5^th ^to 85^th ^percentile BMI; At-risk for overweight = 85^th ^to 95^th ^percentile BMI; Overweight = ≥95^th ^percentile BMI.

^3 ^Sample sizes vary slightly due to missing data.

^4^Significance testing based on chi-square test for linear trend.

^5 ^Frequency and sample size not reported due to confidentiality of subjects (n < 5).

### Utility of Height and Weight Screening

Overall, about half of the students reported knowing their body weight prior to screening. Among males, 61.2% of healthy weight, 50.0% of at-risk for overweight, and 52.9% of overweight students reported prior knowledge of weight, and among females, 53.9% of healthy weight, 52.2% of at-risk for overweight, and 45.2% of overweight students reported prior knowledge of weight. With the exception of females who were at-risk for overweight, fewer than half of all students reported regularly tracking their weight. Overall 58.9% of students reported it was useful to learn their height and weight values; the percentage did not vary by whether or not the student reported knowledge of their weight prior to screening, by age or gender.

### Behavioral Intention Reported After Learning Measured Height and Weight

Students were queried regarding how likely they were to engage in various activities based on learning their height and weight. Overall, the intentions most frequently reported by all students were the intention to increase physical activity (53.3%) and to eat more fruits and vegetables (52.3%) (Table 4). The intentions least frequently reported by all students were the intention to take diet pills or herbal supplements (6.5%) or to visit a weight loss clinic (10.6%).

**Table 4:** Percentage of students indicating intention to engage in weight management activities after learning height and weight, ^1^by weight status category^2 ^(%)

		**Males**				**Females**			
	**Overall^3^** **% (n)**	**Healthy Weight** **% (n)** **n = 253**	**At-risk for overweight** **% (n)** **n = 62**	**Overweight** **% (n)** **n = 85**	**P for trend^4^**	**Healthy Weight** **% (n)** **n = 244**	**At-risk for overweight** **% (n)** **n = 69**	**Overweight** **% (n)** **n = 62**	**P for trend**

**Clinical**									
Visit pediatrician	31.9(244)	27.2(69)	27.4(17)	44.0(37)	<0.01	29.2(71)	26.8(18)	51.6(35)	<0.01
Visit school nurse	18.5(143)	18.6(47)	14.5(9)	20.4(17)	0.917	18.1(44)	25.0(17)	14.5(9)	0.858
Visit weight specialist or nutritionist	17.0(139)	16.2(41)	09.4(6)	15.5(13)	0.632	15.5(38)	17.4(12)	35.6(22)	<0.01
Visit weight loss clinic	10.6(82)	06.8(17)	08.0(5)	15.5(13)	<0.05	7.4(18)	18.8(13)	26.2(16)	<0.01
**Dietary**									
Eat more fruits and vegetables	52.3(404)	43.4(110)	56.4(35)	68.2(58)	<0.01	46.0(112)	69.6(48)	67.7(42)	<0.01
Diet (restrict food)	20.9(161)	08.0(20)	17.7(11)	45.8(39)	<0.01	13.5(33)	33.3(23)	58.1(36)	<0.01
Skip meals or snacks	19.7(153)	12.2(31)	14.5(9)	37.7(32)	<0.01	13.1(32)	30.4(21)	45.2(28)	<0.01
Take diet pills or herbal supplements	6.5(50)	6.8(17)	-^5^	14.1(12)	-	3.3(8)	7.2(5)	9.8(6)	<0.05
**Activity**									
Watch less TV	18.6(143)	16.0(40)	21.7(13)	27.7(24)	<0.05	15.1(37)	22.1(15)	24.6(15)	0.063
Increase physical activity	53.3(405)	45.0(114)	61.0(38)	76.5(65)	<0.01	43.6(106)	61.7(43)	75.4(47)	<0.01
Participate in a sport or exercise class	39.9(304)	35.5(90)	39.0(24)	67.1(57)	<0.01	31.7(77)	33.9(23)	60.6(38)	<0.01

^1 ^The percentages reflect the percentages of respondents reporting to be "very likely" or "slightly likely" on a 5-point Likert scale.

^2 ^Weight status definitions based on CDC 2000 Growth Reference (16):: Healthy weight = 5^th ^to 85^th ^percentile BMI; At-risk for overweight = 85^th ^to 95^th ^percentile BMI; Overweight = ≥95^th ^percentile BMI.

^3 ^Sample sizes vary slightly due to missing data.

^4^Significance testing based on chi-square test for linear trend.

^5^Sample size too small to report (n < 5)

Compared to healthy-weight students, a higher frequency of at-risk for overweight or overweight students reported the intention to perform weight-management related activities (Table 4). Among overweight students, visiting the pediatrician was the most commonly reported clinical intention: 44.0% of overweight males and 51.6% of overweight females planned to do so. Additionally 8.0% of at-risk for overweight and 15.5% of overweight males, and 18.8% of at risk for overweight and 26.2% of overweight females indicated their intention to visit weight loss clinics. And 17.7% of at-risk for overweight and 45.8% of overweight males, and 33.3% of at-risk for overweight and 58.1% of overweight females intended to go on a diet. Eating more fruits and vegetables was the most commonly reported dietary behavior, with 68.2% of overweight males and 67.7% of overweight females indicating that they intended to do so. Increasing physical activity was the most commonly reported activity behavior: 76.5% of overweight males and 75.4% of overweight females.

The intention to perform the eleven weight management activities were subjected to Cronbach's alpha analysis utilizing two different approaches. The Cronbach's alpha based on the five point Likert scale responses to the 11 items was .82 (n = 788) and could not be improved by eliminating any of the items. After dichotomizing the items into likely versus not likely, Kuder-Richardson 20 of .87 (n = 788) was obtained and could not be improved by eliminating any of the items. Overall, twenty-eight percent of the subjects did not report being likely to engage in any of the 11 activities.

After controlling for age, gender, and race/ethnicity, compared to healthy-weight students, students who were either at-risk for overweight or overweight were, in general, significantly more likely to report the intention to perform weight-management related activities (Table 5). Overweight students were approximately twice (95% CI = 1.3, 3.1) as likely to report their intention to visit a medical doctor and three times (95% CI = 1.6, 5.4) as likely to report their intention to visit a weight loss clinic. At-risk for overweight and overweight students were 2.1 times (95% CI 1.3, 3.3) and 2.7 times (95% CI = 1.7, 4.1) as likely, respectively, to report intention to eat more fruits and vegetables. Compared to health weight students, at-risk for overweight and overweight students were 3.2 times (95% CI = 1.9, 5.4) and 8.9 times (95% CI = 5.4, 14.6) as likely to report their intention to diet. Additionally, at risk for overweight and overweight students were 2.0 times (95% CI = 1.1, 3.3) and 4.7 times (95% CI 2.9, 7.5) as likely to report the intention to skip meals or snacks. At risk for overweight and overweight students were also 2.2 times (95% CI 1.4, 3.4) and 4.3 times (95% CI 2.7, 7.0) as likely to report the intention to increase physical activity. And overweight students were 1.8 times (95% CI 1.1, 3.0) as likely to report intention to watch less television compared to healthy weight students.

**Table 5:** Adjusted Odds ratio for behavior intentions reported after learning measured height and weight

Independent Variable	Visit medical doctor OR (95%CI)	Visit School Nurse OR (95%CI)	Visit weight specialist or nutritionist OR (95%CI)	Visit weight loss clinic OR (95%CI)	Eat more fruits and vegetables OR (95%CI)	Diet (restrict food) OR (95%CI)	Skip meals or snacks OR (95%CI)	Take diet pills/herbal supplements OR (95%CI)	Watch less TV OR (95%CI)	Increase physical activity OR (95%CI)	Participate in a sport or exercise class OR (95%CI)
**Gender**											
Male											
	1	1	1	1	1	1	1	1	1	1	1
Female	1.0 (0.7, 1.5)	1.0 (0.7, 1.5)	1.0 (0.7, 1.5)	1.3 (0.8, 2.3)	1.1 (0.8, 1.5)	1.8 (1.2, 2.7)	1.3 (0.9, 2.0)	0.7 (0.4, 2.4)	0.8 (0.6, 1.3)	0.9 (0.7, 1.3)	0.8 (0.6, 1.1)

**Weight status^1^**											
Healthy weight	1	1	1	1	1	1	1	1	1	1	1
At-risk for overweight	0.8 (0.5, 1.3)	1.0 (0.6, 1.7)	0.5 (0.3, 1.0)	1.7 (0.9, 3.4)	2.1 (1.3, 3.3)	3.2 (1.9, 5.4)	2.0 (1.1, 3.3)	0.9 (0.4, 2.4)	1.2 (0.7, 2.2)	2.2 (1.4, 3.4)	
Overweight	2.0 (1.3, 3.1)	0.8 (0.5, 1.4)	1.9 (1.1, 3.1)	3.0 (1.6, 5.4)	2.7 (1.7, 4.1)	8.9 (5.4, 14.6)	4.7 (2.9, 7.5)	1.9 (0.9, 3.9)	1.8 (1.1, 3.0)	4.3 (2.7, 7.0)	1(0.7, 1.6) 3.2 (2.1, 4.9)

**Ethnicity**											
Non-Hispanic white	1	1	1	1	1	1	1	1	1	1	1
Non-Hispanic black	5.4 (3.6, 8.2)	4.2 (2.6, 7.0)	6.4 (3.6, 11.5)	6.2 (3.1, 12.3)	2.5 (1.7, 3.6)	2.5 (1.6, 4.1)	2.5 (1.6, 4.1)	3.0 (1.4, 6.4)	2.3 (1.5, 3.7)	1.5 (1.0, 2.1)	2.3 (1.6, 3.4)
Hispanic	3.5 (2.0, 6.0)	2.3 (1.2, 4.6)	6.6 (3.3, 13.2)	1.1 (0.4, 3.7)	2.0 (1.2, 3.3)	2.1 (1.1, 4.0)	1.8 (1.0, 3.5)	1.6 (0.5, 4.9)	1.2 (0.6, 2.4)	1.4 (0.8, 2.4)	2.3 (1.4, 3.8)
Asian	1.8 (0.9, 3.5)	2.0 (0.9, 4.2)	4.3 (2.0, 9.7)	3.2 (1.1, 9.1)	1.8 (1.0, 3.1)	1.3 (0.6, 3.1)	2.2 (1.1, 4.7)	0.9 (0.2, 4.3)	1.6 (0.8, 3.4)	1.6 (0.9, 2.8)	1.6 (0.9, 3.0)

**Age**	0.9 (0.8, 1.0)	1.1 (1.0, 1.3)	0.9 (0.8, 1.1)	0.9 (0.7, 1.1)	0.8 (0.7, 0.9)	0.9 (0.7, 1.0)	0.9 (0.8, 1.1)	0.8 (0.6, 1.0)	0.8 (0.7, 0.9)	1.0 (0.9, 1.1)	1.0 (0.8, 1.1)

^1 ^Weight status definitions based on CDC 2000 Growth Reference (16):: Healthy weight = 5^th ^to 85^th ^percentile BMI; At-risk for overweight = 85^th ^to 95^th ^percentile BMI; Overweight = ≥95^th ^percentile BMI

Some differences by race/ethnicity and by gender in reported intentions following learning height and weight were seen. For most behavioral intention questions, non-white racial/ethnic groups were considerably more likely to report the intention to perform the behavior compared to non-Hispanic whites. In contrast, gender was a significant predictor of only one of the twelve weight-management behavioral intentions, with girls almost twice as likely as boys to report that they planned to diet upon learning their height and weight (OR = 1.8 95% CI 1.2, 2.7).

## Discussion

The purpose of this study was to evaluate school-based weight screening from the perspective of the students' perceived comfort, utility, and impact of screening on reported behavioral intentions. With the exception of overweight students, particularly females, the majority of middle-school aged students we surveyed felt comfortable with weight screening and most felt that privacy was sufficient. The high rate (61.3%) of overweight female students reporting dissatisfaction with the level of privacy afforded students suggests the need for protocols that further increase privacy. Although the weight screening was not conducted in a private room as is preferable [7,19], the creation of a private environment by measuring weight in a corner of the large school gymnasium seems to have provided adequate privacy for most students. Students reported that learning their height and weight values was useful. Students who were at-risk for overweight and overweight were more likely to report the intention to perform weight-management related behaviors, both potentially beneficial and potentially harmful. Interestingly, no differences were observed by age group, and only intention to diet differed by gender, with girls more likely to plan to diet than boys.

Only 2% of students reported that weight screening should not be conducted at all, suggesting that most students understand the value of the screening activities. However, it is noteworthy that when given the opportunity to select locations for weight screening, 78.2% of students did not identify physical education class, 85.3% did not identify the school nurse's office, and 69.4% did not identify either of these school venues as an appropriate location. Given its universality in clinical settings, it is not surprising that most students felt that weight should be measured at the doctor's office with a much smaller percentage identifying PE class or the nurse's office as a screening location. This discrepancy was observed particularly among for overweight students. Overweight students may deem the doctor's office as appropriate because it is where they are accustomed to being weighed and measured, or it may be preferred because it provides a higher level of privacy and follow-up. Though implied with the setting, we did not ask student to indicate *who *they thought should actually perform the screening. Ikeda *et al *suggested weight screening should be conducted by school nurses (trained health professionals) in a caring and sensitive manner [15].

Approximately half of all students reported knowing their height and weight prior to the screening, and fewer than half of all students reported regularly tracking their height and weight. These findings suggest that sharing height and weight values may be of use to students if, in fact, knowledge of weight assists in the initiation of safe weight-control-related behaviors.

In response to questions regarding their weight control intentions following learning of their height and weight, students most frequently reported that they planned to increase physical activity and to eat more fruits and vegetables, behaviors consistent with current dietary recommendations [26]. However, our results also identified intentions to initiate potentially risky weight control-related behaviors among students of all weight categories, and particularly among at-risk for overweight and overweight students. These behaviors, such as restricting their diet or increasing their physical activity if extreme, have the potential to become unhealthful habits. Although only a small percentage (6.5%) of students reported the intention to perform a potentially unhealthy behavior such as the use of diet pills and herbal supplements, the percentage of students reporting such plans was higher among the students who screened at-risk for overweight and overweight. Behavior change theory suggests that reporting the intention to perform a behavior is predictive of actually performing the behavior, but, of course, it does not ensure its occurrence. In studies that have attempted to directly estimate usage, the prevalence of diet pill use was 2.4% in a study of middle school students [27] and at 7.9% in a study of high school students [28]. Other studies have found that compared to non-overweight students, overweight adolescents use unhealthy weight management strategies more frequently [29] and are less likely to engage in healthier weight management strategies such as healthy eating and physical activity [30]. Although a higher percentage of students in this study overall reported the intention to perform healthy rather than unhealthy behaviors, relatively high numbers of students planning unrecommended weight control measures suggest substantial opportunities for weight-management education. Whether the higher frequencies of behavior intentions reported among at-risk for overweight, overweight, non-white race and ethnic groups resulted in higher levels of these actual behaviors should be explored.

Several limitations of the current research are noteworthy. First, although the survey was completed immediately after weight screening, the students were given height and weight information only, not their weight status category. Knowledge of their weight status classification might have altered their responses to the survey questions. There were also limitations associated with our assessment of the utility of the height and weight screening. Because of the logistics of the study in which height and weight were measured prior to the survey, students were asked to report whether they had knowledge of their body weight after screening. Additionally, the reported knowledge of body weight was not validated (students were not asked to report what their body weight actually was) thus students were reporting whether they *think *they know their body weight rather than whether they actually do. Given that reported usefulness of learning body weight did not differ according to reported knowledge, it is likely that many students who reported knowing their body weight actually did not. Second, whereas immediate reaction to screening provides important information, delayed responses after weeks or even months might differ and could reflect other aspects of comfort. Third, despite training, error in the measurement of individual students' height and weight is possible. The study took advantage of a real-world circumstance in which data collectors were already collecting and recording height and weight data for a school-wide surveillance system. Although trained on standardized techniques, PE teachers, as lay data collectors, may not have achieved research-level accuracy or reliability. However effects of this error would be expected to be random rather than systematic. Generalizability of the survey's findings is of potential concern. Students in the study population attended schools headed by administrators who had prior successful collaborations with the research team and were willing to participate in this study. However, we have no reason to believe that students attending the research study schools are unique according to measured behaviors. Also, most students were accustomed to having their height and weight measured annually in physical education class and, therefore, their responses may not reflect the impressions of students attending schools just initiating weight and height screening. Finally, despite our large sample size, some of the weight management behaviors we studied are relatively rare and thus our ability to identify differences was limited.

## Conclusion

Overall, our results showed that mixed weight middle school students were reasonably comfortable with weight screening, found the height and weight information from the screening somewhat useful, and reported intentions to initiate appropriate weight control measures. However, among overweight and at risk of overweight students, our results showed greater discomfort of screening, as well as heightened intentions to participate in potentially risky weight control behaviors. The variation of comfort level with weight screening across weight status categories may reasonably fuel debate. Some health professionals may argue that the discomfort experienced by at risk for overweight and overweight students is reason not to screen. Others may posit that weight screening has value and comfort and privacy concerns can be addressed. It is important that practitioners understand school-based healthy weight initiatives, so that they are poised to manage patients seeking additional support. With the broad interest in school-based weight screening, the practice is likely to become widespread. Given the higher level of discomfort among overweight students and the relatively high frequency of intent to initiate some potentially inappropriate dieting behaviors, more attention to privacy and education about appropriate weight management strategies in school settings is warranted. Further evaluation is needed to understand how to increase students' comfort level at the time of the screening and to determine whether students' negative perceptions of the screening persist after the event or moderate with repeated screening. Whether behavioral intention translates into action is a key, yet untested, argument for school-based weight screening.

## Competing interests

The author(s) declare that they have no competing interests.

## Authors' contributions

KK assisted with conceptualization, implementation and analysis of the study and served as project manager. VRC assisted with the conceptualization, implementation and approval of the study. KP assisted with the conceptualization of the study. RMcG assisted with the implementation and approval of the study. RH assisted with the data analysis of the study. A Must assisted with the overall creation, implementation and data analysis. All authors read and approved the final manuscript.

## Pre-publication history

The pre-publication history for this paper can be accessed here:


